# Evaluation of a Physical health plan for people with psychosis: a protocol for a quality improvement study

**DOI:** 10.1186/s40814-019-0396-7

**Published:** 2019-01-15

**Authors:** Julie Williams, Nick Sevdalis, Fiona Gaughran

**Affiliations:** 1 Health Service and Population Research Department, Institute of Psychiatry, Psychology and Neuroscience, King’s College London, De Crespigny Park, London, SE5 8AF UK; 2 Centre for Implementation Science, Institute of Psychiatry, Psychology and Neuroscience, King’s College London, London, UK; 3 Psychosis Studies, Institute of Psychiatry, Psychology and Neuroscience, King’s College London, London, UK; 4 National Psychosis Service, South London and Maudsley NHS Foundation Trust, London, UK

**Keywords:** Psychotic disorders, Mental health services, Quality improvement, Physical health

## Abstract

**Background:**

People with serious mental illness (SMI) have poorer physical health and reduced lifespans compared to the general population. Reasons for this are complex, but one important area is the identification and treatment of physical health conditions. In England, services are structured into primary and secondary care; physical and mental secondary health services are separate. This often leads to a lack of co-ordination of care, with people missing the screening and treatment they need. People with SMI may find accessing physical health services more difficult due to the impact of their symptoms and/or a lack of social support. When they do access physical care, there may be ‘diagnostic overshadowing’ where physical health concerns are put down to a mental health diagnosis. Creating tools to support people with SMI to assume more control of their physical health may help to ameliorate some of these problems. The aim of this study is to evaluate the use of a service user-held Physical health plan (PHP) for secondary mental health service users to determine whether its use increases uptake of physical health services.

**Methods:**

We will undertake a pilot quality improvement (QI) study to test the use of the PHP. The development of the PHP is described. A Theory of Change (ToC) has been developed which we will test to understand how the PHP is used, using focus groups at the beginning of the study. We will then pilot the use of the PHP for 6 months in two community mental health teams to find out how people use it, what actions are taken, and if it increases uptake of physical health care. We will use the RE-AIM implementation framework to guide the evaluation. After the pilot, we will undertake interviews with service users and clinical staff to elicit their views on using the PHP.

**Discussion:**

This study uses QI methodology and an implementation framework to test a novel intervention for people with SMI. If successful, the intervention will support people with SMI to access physical health services. The study will inform the design of a larger-scale definitive RCT.

**Trial registration:**

ClinicalTrials.gov Identifier: NCT03178279. Registered date: 05/06/2017

**Electronic supplementary material:**

The online version of this article (10.1186/s40814-019-0396-7) contains supplementary material, which is available to authorized users.

## Background

People with serious mental illness (SMI) such as schizophrenia, bipolar disorder, and major depressive disorder have worse physical health than the general population, and research has consistently shown that they die younger [1]. The causes of this are complex and include side effects of psychotropic medication, very high smoking rates, sedentary lifestyles, and poor diet, along with inequalities in healthcare provision and in wider determinants of health [2].

A key inequality in healthcare provision in this cohort is the less assertive treatment of physical health conditions, as well as fragmented physical health care across primary care and secondary mental health services [3, 4]. Under-recognition of presenting physical health problems in someone with a known mental illness, so-called diagnostic overshadowing, compounds the situation [5]. The problem is global; similar diagnostic overshadowing has been reported internationally [6] including in the USA [7] and Australia [8].

Although the UK National Institute for Health and Care Excellence (NICE) guidelines state that the physical health of people with psychosis should be monitored by primary care, this does not consistently happen in practice [9]. Primary care staff may not always be confident working with people with SMI [10]. Reciprocally, secondary mental health staff often feel under-confident in identifying and managing physical health problems [11]. Although the UK has primary and secondary care incentivisation strategies for physical health screening and intervening as set out in the NICE guidelines above, there is no nationally agreed template for services to identify and manage physical health issues [12]. Sharing information between primary and secondary care remains difficult [13], although one template for use by primary care has been developed [9]. There are also no agreed effective interventions to improve the physical health of people with SMI, rather the same protocols as applied to the general population are used. This is starting to be addressed by initiatives such as Equally Well which aims to bring organisations together such as NHS Trusts and third sector organisations to develop resources and good practice across the UK (https://www.centreformentalhealth.org.uk/campaigns/equally-well) and the Healthy London Partnership which provides helpful resources (https://www.healthylondon.org/our-work/mental-health-transformation/stolen-years/).

To date, there is little understanding of how people with SMI experience their physical health and the support they would like with it. The barriers to self-managing physical health identified by mental health service users include the debilitating nature of mental illness, having a poor understanding of physical health, the experience of stigma from medical staff, and social isolation [14]. Self-management can improve health outcomes in this cohort [15], and tools such as Wellness Recovery Action Plans (WRAP), which have been developed with resources to be completed by the individual for people to self-manage their mental health, can be helpful in supporting personal recovery in mental health [16].

The aim of this study is to address some of the gaps identified in how people with SMI manage their physical health. Specifically, we ask whether the use of a service user-completed Physical health plan (PHP) by people with SMI using community mental health services increases their autonomy and uptake of physical health services.

## Methods

### Design

This is a multi-phase study using the Medical Research Council (MRC) framework for the evaluation of complex interventions [17], incorporating quality improvement principles to evaluate the use of the PHP and any subsequent actions taken. The objective of the study is to design and evaluate a PHP for use by service users of community mental health teams. The MRC framework recommends [18] four phases in the study design: (1) ‘pre-clinical’, (2) ‘modelling’, (3) ‘exploratory trial’, and (4) ‘definitive trial’. We have already undertaken phases 1 and 2 which are described in the following section. This protocol focuses on phase 3—an exploratory trial of the use of the PHP.

Two phases of the study have been completed already:

1. ‘Pre-clinical’ developmental phase

We undertook focus groups with service users asking about support they have received for their physical health and their views on the PHP. People who had attended a course on understanding psychosis at the SLaM Recovery College were contacted by email with an invitation to attend. Two focus groups were run, each with three participants. Informed consent was obtained from all participants. We found that participants experienced varying levels of support for their physical health from primary and secondary care and there was not always co-ordination between the two. They liked the idea of the PHP as they felt this would help them to take more control of their physical health needs and hoped it would be a helpful tool in the integration of their care.

We also undertook a service gap analysis with community mental health team leaders working with people with SMI in the South London and Maudsley NHS Foundation Trust (SLaM) to identify gaps in physical health service provision. A questionnaire was designed to find out how physical health needs were identified and addressed in the team, who was involved, and what the challenges were. Twenty team leaders were contacted, and 13 (65%) completed the questionnaire. We found that all participants understood the importance of physical health and wanted to support their service users, but this could be difficult due to the other priorities they had.

The reports on both of these pieces of work are available from the corresponding author on request.

2. Modelling phase—designing the intervention

#### Development of the PHP

The PHP was designed by research team members (JW, FG) using information from available resources including:

1. The Bradford Mental Health Physical Review [9]. This resource was developed to provide a template for the data derived from annual physical health checks undertaken by general practitioners in England for people with SMI and is completed by primary care staff with the aim of ensuring this information is collected.
2. The Rethink Physical Health Check [19]. This was developed as a collaboration between psychiatrists and Rethink Mental Illness, a prominent UK mental health charity, as part of a toolkit of resources developed for health professionals and service users regarding the importance of physical health. It is designed to be completed jointly by the service user and their healthcare worker.
3. The Physical Health Screen of the SLaM NHS Foundation Trust Electronic Patient Journey System. SLaM has an electronic patient note system with templates developed to enter key patient data including responses to physical health screening questions. This is stored in the electronic notes and is not shared with the service user.
4. The Personal Health and Care Screening section used in ‘Integrating Mental and Physical Health: Research, Training and Services (IMPARTS)’, an initiative funded by King’s Health Partners (KHP) to integrate mental and physical healthcare. IMPARTS have developed a screening system for people accessing physical health services for anxiety and depression [20]. When data requiring action are identified, the clinical team are alerted. IMPARTS has not to date been used in a mental health setting.

Each of these sources has different aims and is not designed to be used solely by service users. They were consulted as they provide information on which physical health conditions are considered key for this population. They rely on clinicians to plan or help plan actions. The PHP is designed to be used by service users with support if required, rather than by staff, so it has a different emphasis and aim.

The wording and content of the PHP was then further refined following two further focus groups with six service users and one session with three service users who were asked to complete the PHP and give feedback.

#### The intervention

##### Rationale

The PHP consists of a series of questions about the individual’s physical health needs, their use of primary care, and their uptake of indicated screening and resultant healthcare actions.

The use of the PHP attempts to address two issues critical for the provision of adequate physical care to people with SMI:

1. Supporting self-management and autonomy for SMI service users in dealing with their health
2. Assisting mental health staff to support SMI service users where needed in accessing physical health monitoring and interventions

The overall aim of the PHP is to increase the uptake of physical healthcare services (e.g. screening, health monitoring) by service users.

##### Materials

The PHP consists of questions regarding physical health needs (e.g. whether the service user has diabetes) and uptake of health checks (e.g. whether the service user has had their blood pressure checked in the last year) and covers physical health areas such as blood pressure, weight, diabetes, smoking, dental care, pain, and health screening. The PHP is designed so that it can be responsive to changes in guidelines and recommended good practice. This will be done by regularly reviewing the evidence and updating as necessary.

##### Procedure

**Fig. 1:**
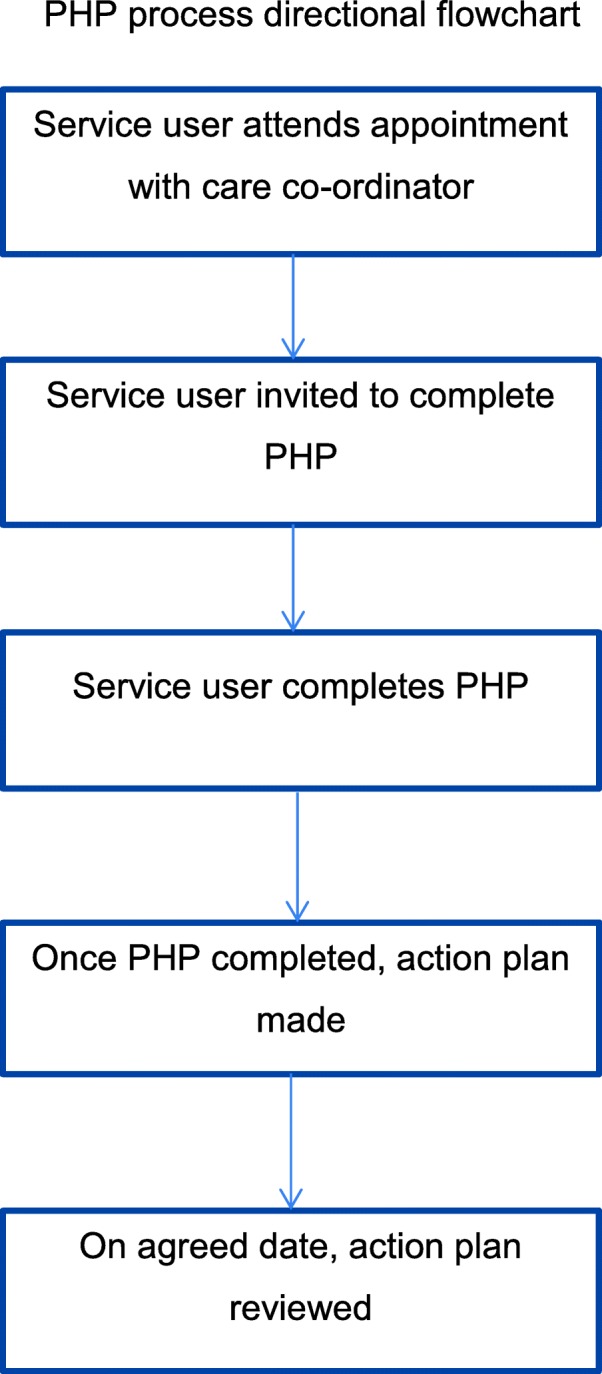
PHP process

The PHP will be completed by the individual service user, supported by their care co-ordinator if needed, which will generate an action plan, shared with the care co-ordinator, to address any gaps or needs identified. The PHP will then be repeated at 4–6 months which will quantify actions taken and remaining unmet need. The process is shown in Fig. 1.

##### Modes of delivery

The PHP will be available in three formats—as a paper version, on an existing electronic database (IMPARTS) and on a new patient-held record (Healthlocker). These are described in more detail later.

##### Locations

The PHP is designed to be used by service users and could therefore be used in any location. We are piloting it in community mental health teams.

The PHP will be completed once and then repeated 4–6 months later.

Tailoring—we will use the pilot to find out if the use of the PHP is different for different people and how much support is required to complete it.

Modifications—we will monitor and describe any modifications that are required.

*Exploratory study of the use of the PHP*

We will undertake an exploratory study of the use of the PHP in two community mental health teams.

#### Conceptual framework of the study

The exploratory study will be guided by a Theory of Change (ToC), and evaluation will be guided by the RE-AIM implementation framework. Both are described below.

##### Theory of Change

**Fig. 2:**
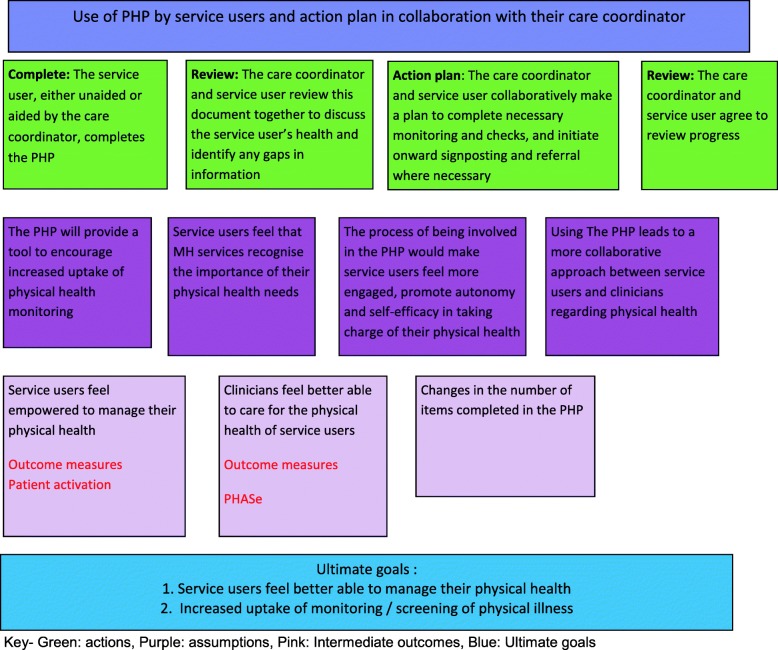
Theory of Change for the present study

A ToC is a way of representing visually both how an intervention is expected to have impact and the assumptions behind it, within a structured diagram [21]. ToCs have been used to clearly show the rationale behind an intervention to help understand why it does or does not work. A ToC clearly shows to all participants the assumptions behind the intervention and can be used to discuss the intervention and formulate its implementation with key participants. We will use a Theory of Change (ToC) of how the intervention works (or not) to guide our study (See Fig. 2 for our ToC).

The ToC will be shared with service users and clinical staff to obtain their input. This approach will allow us to check and ‘validate’ the research team’s assumptions and understanding of how the intervention will work with the people who deliver and attend that particular service. Creating such shared understanding of how a service-level intervention may work in practice is a basic premise of implementation research.

##### Implementation framework

We will use the RE-AIM framework [22] to guide our evaluation. This framework was developed in 1999 and is designed to assist planning and evaluation of interventions and implementation. It is a widely used implementation framework [23] which addresses five key issues in implementation design, as follows:

- Reach—How do I reach the targeted population with the intervention?
- Efficacy—How do I know my intervention is effective?
- Adoption—How do I develop organisational support to deliver my intervention?
- Implementation—How do I ensure the intervention is delivered properly?
- Maintenance—How do I incorporate the intervention so that it is delivered over the long term?

In this study we are using the RE-AIM framework to understand how the PHP is implemented in each team and to understand the implementation challenges.

### Setting

The study will take place in two community mental health teams in SLaM working with people with SMI. These teams work using the Care Programme Approach (CPA) [24]. The CPA is used in England as the framework for secondary mental health services which includes each service user having a named care co-ordinator. Each team works with between 100 and 200 service users.

### Procedure

#### Recruitment process

The exploratory pilot teams which are interested and have the capacity to be involved will be chosen by asking managers in SLaM. All service users using these teams are eligible to take part as long as they are able to give informed consent. The researcher undertaking this study will conduct the focus groups and conduct recruitment. They will not be known to the service users or staff who will be approached for the study.

The exploratory study of the use of the PHP will have three stages: (1) exploratory qualitative stage, (2) 6-month PHP use, and (3) qualitative evaluation stage.

*Stage 1: Initial qualitative work to refine and validate the Theory of Change*

We will undertake focus groups with service users and staff to test out the assumptions outlined in the ToC above. Focus groups are a useful method in developing and strengthening ToCs by enhancing stakeholder engagement and allowing the researcher to better understand the context of the intervention [25].

The focus groups for service users will consist of six to eight service users in each team. As this is an exploratory study, we will keep our inclusion criteria deliberately broad to encourage participation by a broad range of participants with our only inclusion criteria that participants are able to give informed consent. To recruit service users, we will ask staff in each team to give details of the focus group to anyone who meets our inclusion criteria and ask if they would like to participate in the focus group. The researcher will then contact each person and explain the focus group further. All participants will be asked to give informed consent.

The staff focus group will consist of all of the staff in the team to ensure that all staff are aware of the PHP and are able to give their opinion. We anticipate that the number of staff in each team will vary but anticipate teams of between 10 and 20 people. This is important to support staff ‘buy-in’ for the study.

Each focus group will be guided by a semi-structured topic guide. Questions will focus on the process and assumptions outlined in our ToC so that we can find out if these meet the experiences and expectations of the participants. We will outline the process of accessing and completing the PHP and ask staff and service users to comment on this and any difficulties they anticipate and any changes that may need to be made. We will discuss with service users and staff why the PHP has been developed and how we propose it be used, including how the PHP should best be introduced to service users to make it understandable and meaningful. We will also ask about the role of carers in the use of the PHP and incorporate ideas for involving carers into our evaluation. Not all service users want carers to be involved in their care, but when they do, we wish to be able to facilitate this.

We will explain that this is a quality improvement project in which we are piloting the PHP and will explore with staff where the PHP will fit into their existing care pathways, along with ways of working to address implementation issues before starting to use the PHP. As this is a quality improvement (QI) study, we can consider different opinions and trial different routes to implementation. This work will allow us to understand the context in which the PHP will be introduced, to understand the views of key stakeholders towards it, and to optimise its implementability.

*Stage 2: Use of the PHP*

The PHP will be completed by individual service users with support from healthcare staff if required. The PHP will be available on three platforms: one paper-based and two digital, the IMPARTS interface and Healthlocker. Each platform will be available to all service users, and they will be able to choose which platform they use. To develop the digital platforms, the research team had many discussions with digital technology experts including the IMPARTS Team, SLaM IT staff, and the Mindwaves team to develop the infrastructure needed to support the PHP and to design the best platform. The electronic versions of the PHP will be available to participants either on a tablet (IMPARTS), such as an iPad, or a tablet or smartphone (Healthlocker) (Table 1).

**Table 1:** PHP platforms

Platform	Description
Paper copy	Where an electronic version of the PHP is unavailable, or where people do not wish to use the electronic version, they will be offered a paper copy instead which will be uploaded to their electronic notes.
IMPARTS	Integrating Mental and Physical Health: Research, Training and Services (IMPARTS) (https://www.kcl.ac.uk/ioppn/depts/pm/research/imparts/index.aspx) is an initiative funded by King’s Health Partners (KHP) to integrate mental and physical healthcare into research, training, and clinical services at Guy’s and St Thomas’s Trust, King’s College Hospital, Kings College London, as well SLaM. The overall goal of IMPARTS is to improve mental healthcare provision within medical settings across KHP. The IMPARTS package for physical healthcare settings is designed to support clinical teams in providing timely, tailored, evidence-based care to patients presenting at King’s Health Partner’s acute trusts.
Healthlocker	This is an electronic patient-held record which will soon be rolled out across some teams in SLaM. Participants signing up to the Healthlocker system in designated services in SLaM will be invited to complete a PHP, which they may then share with clinicians and their carers.

We will record which available version participants choose as part of the evaluation of the study (these data will inform larger-scale implementation of the PHP).

Depending on the version chosen, a paper version can be given or, for the IMPARTS platform, the PHP will be placed on an iPad, available at the team reception for completion by service users, or, with the Healthlocker platform, the participant may use their smartphone to access the PHP. The data from the completed paper PHPs will be held by the research team and a copy uploaded with the patient’s permission to the electronic patient notes. The IMPARTS PHP version will be stored on the King’s College Hospital server, and the IMPARTS team will supply the research team with this data. The Healthlocker PHP version will be held by the service user in the cloud, and the data will be accessible through the electronic notes, with the service user’s permission.

#### Introduction of PHP to service users

Staff in each participating team will be asked to introduce the PHP to all of the people on their caseload and invite them to complete it. We will make it clear that this is not compulsory. For each person who completes a PHP, informed consent will be obtained. An iPad/tablet will be placed in the reception of each participating team, with paper versions also available, and service user encouraged to complete the PHP whilst waiting for their appointments. If a paper version is used, a copy will be kept by the service user and one given to their care co-ordinator if they agree to this, and this copy will be uploaded on ePJS. If the PHP is completed in the iPad, it will then be emailed to the service user and their care co-ordinator (with permission). For service users who do not have an email address, the care co-ordinator will print a copy for them and ideally give it to them in the appointment or send it to them by post. Where Healthlocker is available, a service user may choose to use the PHP on their smartphone.

If there are gaps in the completed PHP, the service user will be asked to develop an action plan, with support from their care co-ordinator if wanted. As part of the evaluation of the study, the contents of each action plan and progress made with the action plan will be recorded if the participant gives permission for this. Each action plan will be reviewed at 4 months when the PHP is repeated, and any actions taken will be recorded.

One of the researchers will work closely with each of the participating team throughout the exploratory study to provide support for any problems or challenges encountered by staff. These will be logged to ensure a record is kept (as this will inform our implementation analysis and the future implementability of the PHP), and any changes made to procedures also logged.

All trial data will be stored by the research team in locked cabinets and password-protected files, and confidentiality will be maintained at all times. The research team only will have access to the final trial dataset.

This exploratory study will last 6 months.

### Outcomes

In this exploratory study we will evaluate:

1. How many service users are invited to complete the PHP (Reach)
2. How many service users express an interest in taking up that offer (Reach)
3. Which version of the PHP was used (i.e. paper vs digital)
4. How many service users begin a PHP (Reach)
5. How many PHPs are completed fully and breakdown of which items are completed/not completed (Efficacy)
6. The time taken to complete the e-PHP (Efficacy) (measured automatically electronically)
7. Whether the service user needed support from their care co-ordinator to complete the PHP (Efficacy)
8. How many service users complete a repeated PHP (Maintenance)
9. The change in uptake of physical health actions by service users between first and repeat PHP
10. The timescale of any actions taken will be determined using an audit of the Electronic Patient Journey System from the date that the PHP is completed.
11. Patient activation levels using the Patient Activation Measure (PAM) [26]
12. Staff confidence in working with physical health using the Physical Health Attitude Scale for mental health nurses (PHASe) [27]

Outcome data will be collected by one researcher (JW) depending on the version used as follows: the researcher will collect data from clinical staff on their caseload, who has been offered the PHP and who expressed an interest in using it, who began one and who completed it, and who needed support. The researcher will monitor which version of the PHP is used, how many people complete a repeated PHP, and change in uptake of physical health actions. The PAM will be completed when the initial PHP is completed, and the PHASe questionnaire will be completed when researcher starts working with each team.

**Fig. 3:**
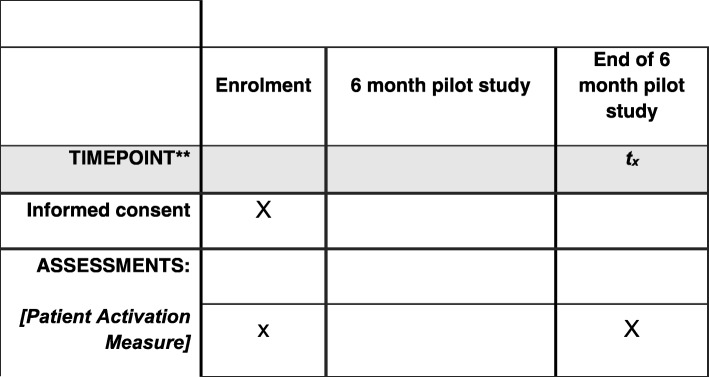
PHP study schedule of enrolment and assessments

Figure 3 shows the PHP study schedule of enrolment and assessments. As this is a QI study, this figure is different to traditional RCT schedules. The SPIRIT checklist can be found in Additional file 1.

We will use the data gained from the study to understand the characteristics of the people who require more support in completing the PHP and the preferences for either the paper or digital versions. We will also capture data on how much training and which format was needed for successful implementation.

*Stage 3: Qualitative evaluation of the use of the PHP*

Interviews will be undertaken with a random selection of service users and clinical staff at the end of the study period. We do not have an estimate of how many people will engage with the PHP. Depending on uptake, we anticipate that 10–20% will be approached and invited to take part in the interview. We do not anticipate more than 20 interviews. The interviews will be semi-structured and ask questions to understand:

- Service user views on using the PHP and the actions they take after completing the PHP
- Staff views on the use of the PHP and the actions taken after the PHP is completed
- What, if any, problems were encountered in completing the PHP

We will attempt to contact service users who declined to complete the PHP to ask their reasons for not wanting to complete it. Informed consent will be obtained for all interviews.

### Patient and public involvement

Involving key stakeholders, particularly service users and clinical staff, is an important part of this project. In the pre-clinical and modelling phases, we involved service users as both facilitators and participants in focus groups and surveyed team leaders. We aim to work with some of the participants in the initial focus groups to help us to monitor and evaluate the project, by setting up a steering group which will meet six monthly over the period of the project and be involved in commenting on the findings of the study and making plans for future implementation. We will work with the steering group to devise a dissemination plan for participants.

### Analysis

#### Quantitative data

We will analyse the use of the PHP using the questions listed above and calculate numbers and percentages for each question. For the PAM and PHASe questionnaires, we will look at change in scores.

#### Qualitative data

We will analyse the interviews after the use of the PHP using thematic analysis to capture the main themes in the use of the PHP. Thematic analysis will be used as it is a method of analysing qualitative data that seeks to identify and make sense of any themes found in the data. We will follow the steps outlined by Braun and Clarke [28].

## Discussion

The information from the exploratory study will help us to understand how people use the PHP, what aspects of it are helpful, and how it is best used and implemented in individual teams and by individual service users. This will therefore help us in designing a larger trial to evaluate effectiveness and to be able to roll out the PHP for use across the Trust and wider across the NHS. People with SMI are known to have poorer physical health than the general population. This has become more widely acknowledged in recent years, and how to address this is a key question [29] with action needed at different levels from societal to individual. The PHP is one method developed to address this. This study aims to evaluate a service user-completed Physical health plan to find out if using it will increase access to physical health services. It is important to understand how new initiatives work at team level to understand if they can be implemented at scale. The use of a Theory of Change and the RE-AIM framework will help us to do this. We will use QI methodologies which will allow us to modify or amend the procedures if this is considered helpful by participants. We will work closely with service users and clinical staff to understand how it is used. This will also allow us to understand if the PHP can be implemented on a larger scale and inform the design of a large scale definitive RCT.

## Trial status

Protocol version number: 13, date: 09.02.2017, recruitment began: December 2017, recruitment will be completed: September 2019.

## Additional files

Additional file 1: SPIRIT 2013 Checklist. (DOC 122 kb)

## Abbreviations

ePJS: Electronic Patient Journey System
IMPARTS: Integrating Mental and Physical Health: Research, Training and Services
KHP: King’s Health Partners
MRC: Medical Research Council
NICE: National Institute of Health and Care Excellence
PHP: Physical health plan
QI: Quality improvement
SLaM: South London and Maudsley NHS Foundation Trust
SMI: Serious mental illness
ToC: Theory of Change
WRAP: Wellness Recovery Action Plans
